# The IL-6 response to *Chlamydia* from primary reproductive epithelial cells is highly variable and may be involved in differential susceptibility to the immunopathological consequences of chlamydial infection

**DOI:** 10.1186/1471-2172-14-50

**Published:** 2013-11-15

**Authors:** Kelly Cunningham, Scott H Stansfield, Pooja Patel, Shruti Menon, Vivian Kienzle, John A Allan, Wilhelmina M Huston

**Affiliations:** 1 Institute of Health and Biomedical Innovation, Queensland University of Technology, Q Block, 60 Musk Ave, Kelvin Grove, QLD 4059, Australia; 2 The Wesley Research Institute, and Wesley Reproductive Medicine and Gynaecological Surgery Unit, 40 Chasley Street, Auchenflower, QLD 4066, Australia; 3 The Wesley Research Institute, The Wesley Hospital, 40 Chasely Street, Auchenflower, QLD 4066, Australia

**Keywords:** *Chlamydia*, Infertility, IL-6, Immunopathology

## Abstract

**Background:**

*Chlamydia trachomatis* infection results in reproductive damage in some women. The process and factors involved in this immunopathology are not well understood. This study aimed to investigate the role of primary human cellular responses to chlamydial stress response proteases and chlamydial infection to further identify the immune processes involved in serious disease sequelae.

**Results:**

Laboratory cell cultures and primary human reproductive epithelial cultures produced IL-6 in response to chlamydial stress response proteases (CtHtrA and CtTsp), UV inactivated *Chlamydia,* and live *Chlamydia*. The magnitude of the IL-6 response varied considerably (up to 1000 pg ml^-1^) across different primary human reproductive cultures. Thus different levels of IL-6 production by reproductive epithelia may be a determinant in disease outcome. Interestingly, co-culture models with either THP-1 cells or autologous primary human PBMC generally resulted in increased levels of IL-6, except in the case of live *Chlamydia* where the level of IL-6 was decreased compared to the epithelial cell culture only, suggesting this pathway may be able to be modulated by live *Chlamydia*. PBMC responses to the stress response proteases (CtTsp and CtHtrA) did not significantly vary for the different participant cohorts. Therefore, these proteases may possess conserved innate PAMPs. MAP kinases appeared to be involved in this IL-6 induction from human cells. Finally, we also demonstrated that IL-6 was induced by these proteins and *Chlamydia* from mouse primary reproductive cell cultures (BALB/C mice) and mouse laboratory cell models.

**Conclusions:**

We have demonstrated that IL-6 may be a key factor for the chlamydial disease outcome in humans, given that primary human reproductive epithelial cell culture showed considerable variation in IL-6 response to *Chlamydia* or chlamydial proteins, and that the presence of live *Chlamydia* (but not UV killed) during co-culture resulted in a reduced IL-6 response suggesting this response may be moderated by the presence of the organism.

## Background

*Chlamydia trachomatis* is the most prevalent sexually transmitted bacterial infection worldwide [1]. The infection is frequently asymptomatic and can result in the development of infertility in 4-20% of infected women [2]. The infertility results from fallopian tube damage such as scarring or complete tubal blockage. Several different models have been proposed to explain the mechanism of immunopathology. The hypersensitivity to chlamydial HSP60 model is supported by evidence including high titres of antibodies against this protein being found in the serum of women with tubal infertility, and immunopathological reactions to doses of this protein in animal models subsequent to chlamydial infections [3-6]. However cHSP60 antibodies found in human sera have been reported to cross-react with other bacterial species indicating the assays may not necessarily have detected *Chlamydia* specific antibody titres [7]. Also, a molecular mimicry model where antibodies to cHSP60 cross-react against human HSP60 has been proposed, however recent data does not support that cHSP60 antibodies cross react with human HSP60 [8]. The ‘cellular paradigm’ model is supported by a number of studies, this model proposes that the reaction of the primarily infected epithelia in the reproductive tract during chlamydial infection determines the disease outcome in each individual [9]. A study using an *ex vivo* fallopian tube organ culture model study demonstrated that IL-1(α and β) were produced by fallopian tube epithelial cells after live chlamydial infection [10]. The damage required live *Chlamydia* and did not require immune cell infiltrates as these were not present in this model [10]. One implication of this study is that the induction of IL-1 and subsequent pathology may involve chlamydial effectors that are exported from the *Chlamydia* vacuole. However, none of these models has been unequivocally validated and it remains uncertain why some (but not all) women develop reproductive tract damage as a consequence of chlamydial infection.

In order to further understand the chlamydial antigens which may be involved in the development of disease we have previously examined the serum immunoglobulin response to chlamydial stress response proteases in women with chlamydial infertility or treated infections with no reported disease pathology [11]. Of particular interest to us were two stress response proteases which have been detected both inside the chlamydial inclusion and in the host cell, CtHtrA and CtTsp [12-14]. Whilst the presence or absence of serum antibodies against these full length proteins (detected by Western blots) was not significantly different between participant cohorts (infertile or treated infections), there was a trend towards different IgG subtype serological responses depending on the participant disease cohort (CtTsp was recognised by IgG3 in the single resolved infection cohort only) [11]. One possible explanation for the observation of differential immunoglobulin subclass responses is that these proteins are associated with the different immune responses which results in different disease outcomes.

This project aimed to investigate the innate and adaptive cellular responses to these two proteases and chlamydial infections to test the hypothesis that they may be antigens which are involved in driving the initial innate pathological response to *Chlamydia.* In both laboratory model cell cultures and primary reproductive cell culture of epithelia or mononuclear cells we observed induction of IL-6 in response to stimulation with these proteins or with *C. trachomatis*. Interestingly, when lab models of mononuclear cells and reproductive epithelia cells were co-cultured we observed that the IL-6 response to live *Chlamydia* was reduced compared to the epithelial cell cultures only (for HeLa and Ishikawa, but not HEp-2). This reduction did not occur for the individual proteins or UV-killed *Chlamydia*. The amount of IL-6 produced from primary reproductive epithelia varied greatly between different participants. Combined these data imply that amount of IL-6 produced from reproductive epithelia during the *Chlamydia* infection may a key factor for the disease outcome in women.

## Methods

### Culture of laboratory cell lines

Human derived cell lines which are commonly used for *Chlamydia* culture experiments were used, including HeLa (cervical carcinoma cell line), Ishikawa (endometrial adenocarcinoma with glandular properties [15]), Ecc-1 (endometrial carcinoma cell line with luminal properties [16]), and HEp-2 (male epidermoid laryngeal carcinoma cell line). HEp-2, HeLa, and Ishikawa were cultured in DMEM (Gibco) containing 10% foetal calf serum, streptomycin (0.1 mg ml^-1^) and gentamycin (0.05 mg ml^-1^), at 37°C 5% CO_2_. A human leukocytic mononuclear cell line, THP-1, was also used. Ecc-1 and THP-1 cells were cultured in RPMI (Gibco) containing 10% foetal calf serum, streptomycin (0.1 mg ml^-1^) and gentamycin (0.05 mg ml^-1^), at 37°C 5% CO_2_. Mouse McCoy cells and mouse macrophages RAW264.7 were cultured in DMEM containing 5% FCS, streptomycin (0.1 mg ml^-1^), and gentamycin (0.05 mg ml^-1^), in 5% CO_2_ at 37°C.

### Preparation of Chlamydia

*C. trachomatis* L2 (strain 434/Bu/ATCC: VR-902B) was cultured using routine methods [12]. Ultraviolet (UV) irradiated *Chlamydia* was prepared by placing an aliquot in wells of a 48-well culture plate and placing the plate 4 cm from a UV light source for 40 min. Samples were then tested for inactivity by culturing on HEp-2 cell monolayers.

### Preparation of chlamydial Tsp and HtrA

Purified recombinant CtTsp and CtHtrA were used as previously described [11]. Purified recombinant CmTsp and CmHtrA were generated for the purposes of this study. The methodology was essential the same as that previously described for CtTsp and CtHtrA [11]. The coding sequences for the proteins was generated by PCR and cloned into the pET22b vector (Novagen) by restriction enzyme digest using *Escherichia* (*E*.) *coli* JM109 cells. Primers used were: CmTsp Rev 5′ GCCTCGAGTTGTGCGGGAGTCTTAATGAAGTTTGC 3′, CmTsp Fwd 5′ GCGGATCCGTCAGCCCCCCTCCGACAACAAGATG 3′, CmHtrA Fwd 5′ GCCCATGGGAATGTTGGGCTATAGTGCGCCAAAGAAAG 3′, and CmHtrA Rev 5′ GCAAGCTTTTCATCAGACTTTAAAACAACGAATCGAATG 3′. Clones were confirmed by restriction enzyme digest and sequence analysis prior to transformation into *E. coli* BL21 for IPTG induced expression of the protein. The protein sequences were cloned in frame with the vector encoded his-tag, and proteins were purified using Talon affinity resin (Clontech, Australia). Protein purity was monitored using SDS PAGE and protein concentration determined using the BCA reagent (SigmaAldrich, Australia), using previously described methods [11].

### Examination of cytokine responses to stimulation of various laboratory cell lines

Epithelial cell lines were seeded at 10 000 cells/well in 96-well plates. In co-culture experiments, THP-1 cells were also seeded at 5000 cells/well. Chlamydial proteins were added at either 2 μg or 10 μg per well, while UV killed *Chlamydia* (L2) and live *Chlamydia* (L2) were added at 5 ul per well (1 × 10^6^ ifu ml^-1^ stock). Supernatants were collected at 96 h after the addition of the stimulants, unless otherwise specified. Samples were frozen at -80°C until ready for assay for cytokine levels by multi-plex bead array (or ELISA in the case of the pathway inhibitor assays). Multi-plex suspension bead array (Bio-Plex) was performed according to the manufacturer’s instructions (Bio-Rad, Australia).

### Primary human reproductive tract cell culture

Primary human reproductive cell culture was conducted on female reproductive tract tissue harvested from consented participants who were undergoing hysterectomy for benign reasons. This study was granted human research ethics committee approval from UC Health Human Research Ethics Committee (Approval number 1101) and QUT Human Research Ethics Committee (Approval number 1100000267). Four participants were included for this investigation and were included in the study due to their low likelihood of a previous history of chlamydial disease, all were undergoing benign hysterectomy. The participants had an average age of 54 years (45–73), none were current smokers, all self-reported to have never had a sexually transmitted infection, all self reported to have never experienced any fertility problems, ectopic pregnancy or pelvic inflammatory disease, only one was currently using contraceptive (QUTPRT05 (monofeme 24)), and three of the four had less than five sexual partners in total.

Isolated endocervical and endometrial epithelia tissues using scalpel shaving into fresh DMEM with 0.2% collagenase D (SigmaAldrich, Australia). The tissue was chopped into fine pieces using a scalpel and further incubated for 10 mins in the DMEM with 0.2% collagenase D. The tissue was then further processed by grinding between two glass slides and incubated at 37°C with constant gentle shaking for single cell suspension. Cells were centrifuged at 1 000 × g for 10 mins at 37°C. the cell pellet was resuspended in DMEM with 0.2% collagenase D for a further 20 mins at 37°C with constant gentle shaking, prior to harvesting the cell and resupension in 4 ml of DMEM containing 2 U/ml DNAse, shaking gently for 2 mins, and then addition of 4 ml of DMEM with 10% FCS to stop DNAse activity. The cells were harvested by Centrifuge at 1000 × g for 10 min at 37°C and resuspended in red blood cell lysis buffer (RBC lysis buffer recipe: NH_4_Cl 0.155 M, NaHCO_3_ 0.012 M, and EDTA 0.0001 M, pH 7.4) for 5 mins at 37°C. The cells were washed in PBS, filtered and again harvested by centrifugation at 1000 × g for 10 mins at 37°C prior to re-suspension in DMEM, 10% FCS, glutamine (4 mmol/L), Gentamicin (0.05 mg ml^-1^) and Strep (0.1 mg ml^-1^) and an aliquot of this suspension was stained with trypan blue and counted using the haemocytometer to allow the cells to be plated. Cells were plated at 10 000 cells per well in 96 well plates for the simulation experiments. Autologous PBMC were used in the co-culture experiments (2000 cells per well), and these were isolated as described below. Cultures were stimulated exactly as described for the laboratory models. The supernatants were harvested 96 h after stimulants added and analysed using Bio-plex bead array.

### Isolation and stimulation of peripheral blood mononuclear cells

Consented voluntary participants provided blood collected into EDTA tubes which were processed for PBMC isolation. The participants consented to allow access to their medical history, and also provided serum samples for serological testing, in order to group them into cohorts, infertile (attending fertility treatment clinic, known not to have any tubal factor infertility), tubal factor infertility (attending fertility treatment clinic, serologically positive to as history of *C. trachomatis* infection by the bio-clone and MEDAC commercial ELISAs), and acute (current urine PCR diagnosed *C. trachomatis* genital tract infection). The infertile cohort was later categorised into *C. pneumoniae* positive and negative cohorts using commercial serological ELISAs for *C. pneumoniae* IgG (MEDAC and Bioclone). The serum from these participants was also used as part of a previously published study [11]. This study has been approved by the QUT HREC approval number 0800000268, Nambour Sexual Health Clinic (EC2809); Ipswich and West Moreton Sexual Health Clinic (10–09); Gold Coast Sexual Health Clinic (200893); Cairns Sexual Health Clinic (HREC/09/QCH/4-554); and Wesley IVF and Gynaecology Clinic (2008/02).

Peripheral blood mononuclear cells were isolated using a Ficoll gradient [17]. Cells were plated at 10 000 or 2000 cells per well (co-cultures) and stimulated and supernatants analysed as previously described.

### Isolation and primary culture of cells derived from murine tissues

Primary culture of mice tissue was conducted from 10 BALB/c mice. This work was approved by the QUT Animal Research Ethics Committee (Approval number 1100000606). Mouse tissues were prepared by surgical harvesting of the caudal lymph nodes and uterine horns from freshly sacrificed naive mice. These caudal lymph nodes and uterine horns were pooled and processed from 10 mice. Single cell suspensions were prepared in a protocol modified from the above human tissue protocol, stimulants added and cytokines measured at 96 h as described above.

### Cell signalling pathway inhibitor assays

In order to determine which signalling pathways were involved in the production of IL-6 in response to chlamydial stimuli, HeLa cells (THP-1 cells), pre-incubated with cell signalling pathway inhibitors, then stimulated with Tsp, HtrA, UV-L2 or L2. The following inhibitors were used in the experiments; Wedelolactone (IKK inhibitor-2; Calbiochem, Australia), PD98059 (broad MEK inhibitor; Calbiochem, Australia), U0126 (specific MEK1/2 inhibitor; Calbiochem, Australia). PD98059 and U0126 were added to wells upon seeding, 24 h prior to addition of chlamydial stimuli. All other inhibitors were added to wells 1 h prior to addition of chlamydial stimuli. Ac-YVAD-CHO was used at a concentration of 10 μM (Garcia-Calvo et al., 1998), and replaced daily. Wedelolactone was used at 2 μM concentration [18]. Both PD98059 and U0126 were used at a concentration of 10 μM. After incubation, 100 ul supernatants were collected for IL-1β ELISA assays at 24 h, while remaining supernatants for IL-6 ELISA assays were collected at 96 h. IL-1β and IL-6 ELISA assays were conducted using commercial kits (Invitrogen, Australia).

### Data analysis and statistics

When appropriate, mean cytokine concentration in control wells (cells cultured with no stimuli) was subtracted from that seen in stimulated cells, so as to account for background cell cytokine levels. Differences between the various treatments were compared using GraphPad Prism software, and p-values were derived from unpaired t-tests.

## Results

### Epithelia cells secrete IL-6 in response to Chlamydia exported proteins which is differentially modulated by co-cultures with THP-1 mononuclear cells

**Figure 1:**
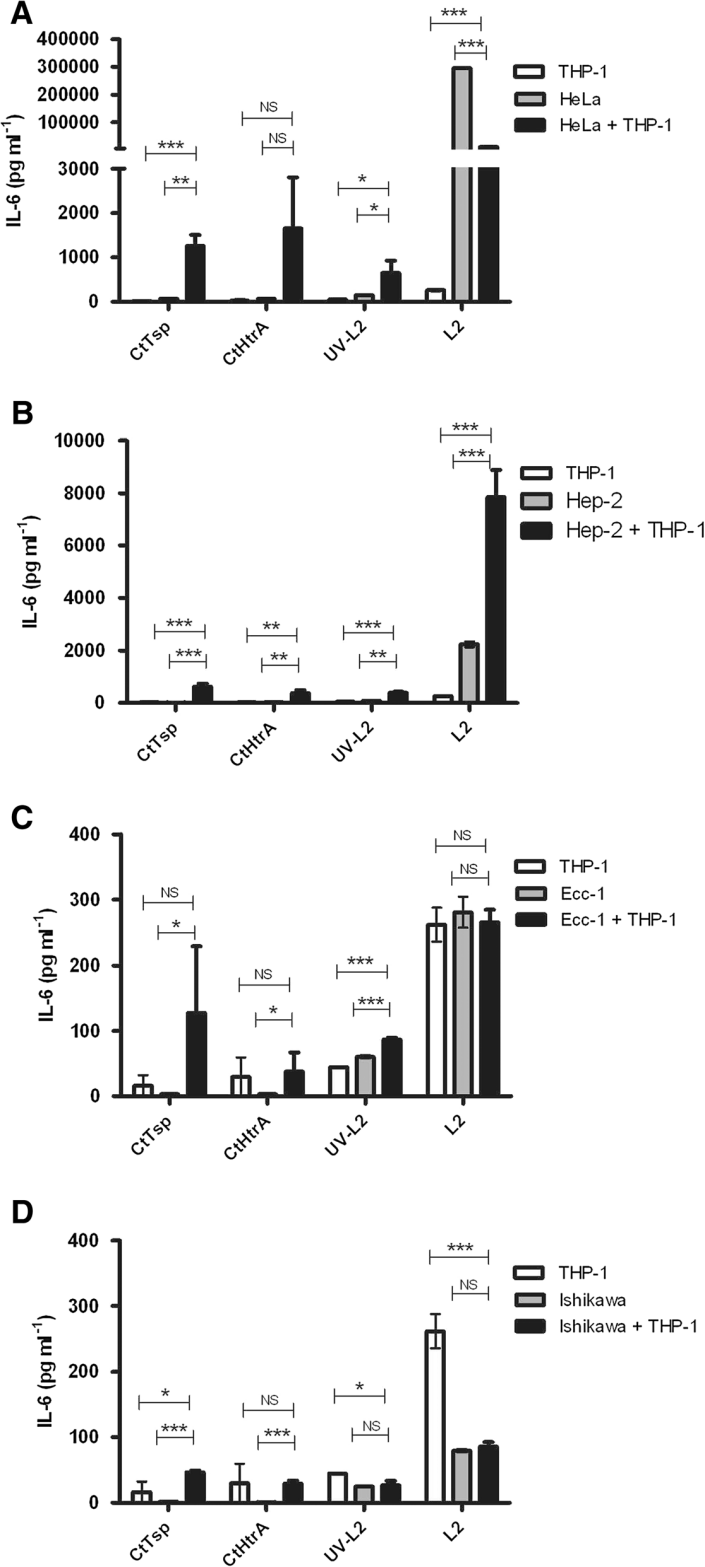
**IL-6 production by laboratory model epithelial cells and THP-1 cells to chlamydial antigens and *Chlamydia*.** The graphs show the supernatant concentration of IL-6 detected at 96 hrs after cells were stimulated with CtTsp, CtHtrA, UV killed *Chlamydia*, or live *Chlamydia* (as labelled on the x-axis). HeLa cells **A)** are of cervical origin, HEp-2 cells are derived from male epidermoid cells **B)**, while Ecc-1 **C)** and Ishikawa **D)** cells are of endometrial origin. Each cell model was also co-cultured with THP-1 (culture conditions indicated by the legend to top left of each graph). The IL-6 values were corrected for mean baseline IL-6 levels seen in unstimulated cells. Unpaired two-tailed t-tests have been performed (n = 3). NS = not significant. * p < 0.05, **p < 0.01, *** p < 0.001.

We conducted an initial experiment using common laboratory model cell cultures, in order to test if the chlamydial stress response proteases CtTsp and CtHtrA have the potential to be PAMPs recognised by human cells. The cells used were HeLa (cervical carcinoma cell line), HEp-2 (male epidermoid laryngeal carcinoma cell line), Ecc-1 (endometrial cancer cell line [16]), Ishikawa (endometrial adenocarcinoma cell line [15]), and THP-1 (human leukemic monocytic cell line). The cells were cultured for four days in the presence of CtTsp, or CtHtrA, or live *Chlamydia*, or UV killed *Chlamydia* (henceforth referred to as stimulants) and the supernatants were analysed for cytokines. All experiments were conducted using THP-1 cells, epithelial cells, or in THP-1/epithelial cell co-cultures (monolayers) (all in triplicate). Figure 1 shows IL-6 detected at 96 h in response to the proteins or *Chlamydia*. Other cytokines examined (IL-1β, IL-10, IL-4, IL-12(p70), IL-13, IFNγ) were not detectable at the 96 h time point in response to any of these stimulants, indicating that IL-6 is part of a sustained response to these PAMPs and *Chlamydia.* Interestingly, some of the cytokines which were not detected at 96 hrs in these experiments have been measured from these cells in response to *Chlamydia* at earlier time points in other published studies [19-21]. chlamydial HSP60 was also tested and found to have a similar cytokine stimulation profile to Tsp and HtrA (Additional file 1: Figure S1) and was consistent with previous reports [22,23].

We observed an increased level of IL-6 in response to CtTsp, CtHtrA, and UV-killed *Chlamydia* stimulation of HeLa and THP-1 co-cultures when compared to either HeLa or THP-1 cells alone. Conversely, co-culture did not enhance the secretion of IL-6 in response to live *Chlamydia*. In fact, the presence of live *Chlamydia* during the HeLa and THP-1 co-culture resulted in a much lower amount of IL-6 secretion, with a reduction of almost 27-fold when compared to IL-6 secretion from mono-culture of HeLa cells with live *Chlamydia* (Figure 1A). In contrast, IL-6 production was markedly increased during the HEp-2 co-culture with THP-1 in the presence of the proteins, UV-killed *Chlamydia*, or live *Chlamydia* treatments compared to individual cell cultures (Figure 1B). Co-culture of Ecc-1 cells with THP-1 resulted in an increase of IL-6 levels compared to Ecc-1 or THP-1 cells alone when stimulated with the chlamydial proteins or UV killed *Chlamydia*. Once again the IL-6 production from co-cultures (Ecc-1 and THP-1) with live *Chlamydia* did not show increased levels compared to either cell line alone (Figure 1C). Very little IL-6 was produced by Ishikawa cells alone, and regardless of the antigen added, the co-culture of Ishikawa cells with THP-1 resulted in comparatively reduced IL-6 levels, similar to the observation for HeLa co-cultures (Figure 1D). The HeLa and HEp-2 cell co-cultures generated far greater levels (10 to 100-fold) of IL-6 in response to the proteins or *Chlamydia* than the Ecc-1 and Ishikawa cells.

Combined this data demonstrates that the different reproductive cell culture laboratory models react differently to *Chlamydia* or chlamydial antigens, and that the interaction between epithelia and live-mononuclear cellular cultures to produce IL-6 appears to be modulated by the presence of live *Chlamydia*. Importantly, the live *Chlamydia* used during this study is the aggressive lymphogranuloma venereum strain L2, therefore the modulation of the IL-6 response in co-culture may be specific to this strain, however the sustained IL-6 production was observed under all conditions using either recombinant protein PAMPs and UV killed or live *Chlamydia* suggesting IL-6 prolonged response is a key component of the innate response to *Chlamydia*. The variable levels in each cell line suggest host specific variability which may indicate an underlying disease susceptibility which we decided to investigate further using primary culture models.

### Primary cultures of female reproductive tract tissues produced IL-6 in response to chlamydial stress response proteases

**Figure 2:**
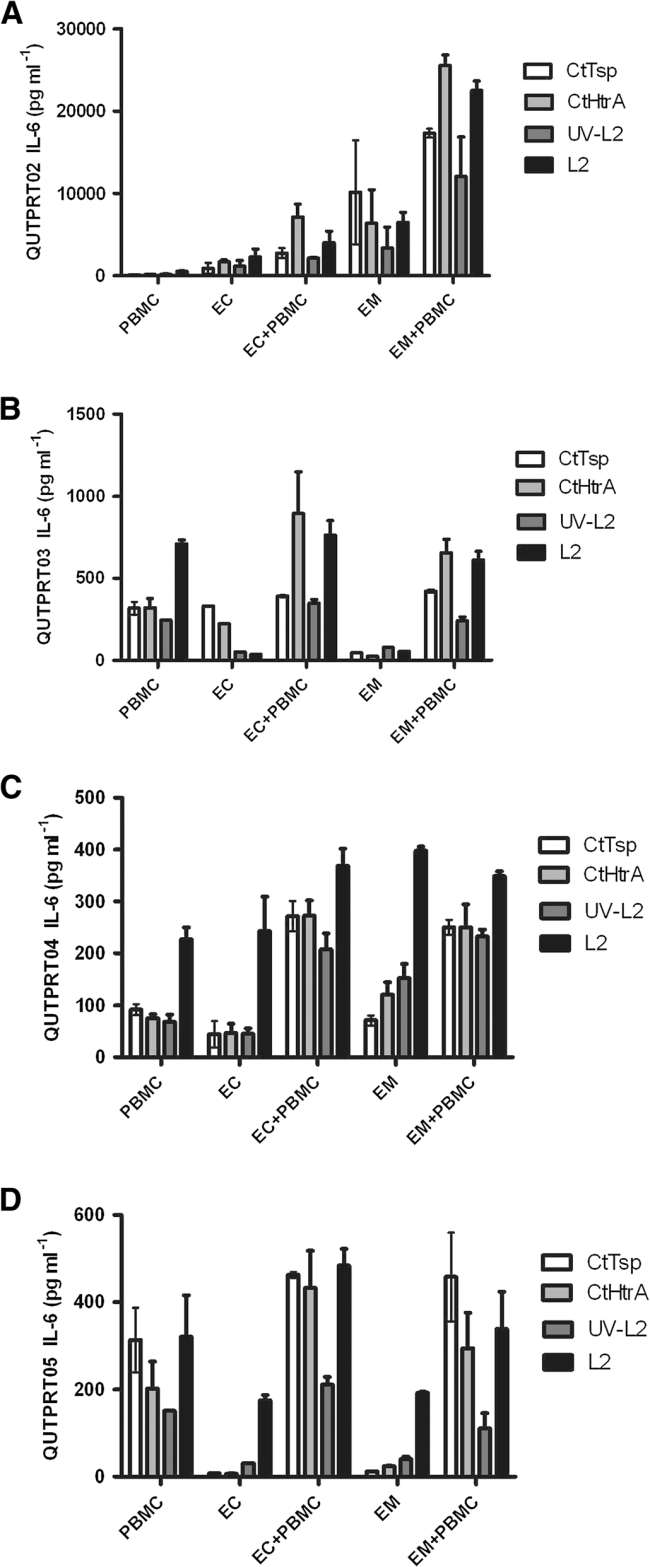
**IL-6 responses in primary cultures of hysterectomy tissue.** IL-6 response detected from primary reproductive cell culture from four separate participants. The graphs show the cellular responses to CtHtrA, CtTsp, live *Chlamydia* and UV-killed *Chlamydia* from endometrial, endocervical, and PBMC primary cultures from human participants. There was a significant variation in the amount of IL-6 secreted; therefore each participant’s data has been shown as separate graphs. **A)** Participant code QUTPRT02, **B)** Participant code QUTPRT03, **C)** Participant code QUTPRT05 and **D)** Participant code QUTPRT05. Each sample IL-6 value has been corrected for mean baseline of IL-6 from unstimulated cells (n = 2 repeats for each sample).

Primary human endometrial and endocervical cells were isolated and cultured with co-cultures of autologous PBMC to monitor cytokine responses to the stimulants (four participants). The cultures were monitored for the presence of IL-6, IL-1β, IL-4, IL-10, IL-12, IL-13 and IFN-γ in the supernatant 96 h after addition of the proteins or *Chlamydia*. As observed for the laboratory cell model experiments presented in Figure 1, IL-6 was detected during all conditions tested. Minor amounts of IL-1β and IFN-γ were detected in some cases, in the range of 0–20 pg/ml and therefore not at clinically relevant levels (data not shown). None of the other cytokines tested were detectable at 96 h (i.e. IL-4, IL-10, IL-12, and IL-13),

The IL-6 levels produced by the epithelia and PBMC varied between ~150 pg/ml and 21 000 pg/ml between different participants, indicating that the IL-6 response varies widely between individuals. Cell isolation protocols, cell numbers cultured, and concentration of proteins added were identical for each condition, as detailed in the Methods. This considerable variation in IL-6 levels means that pooled participant data does not fairly represent the distribution of data observed; hence for clarity and accurate representation of the data, individual participant data has been separately presented in Figure 2. IL-6 levels secreted in response to the chlamydial proteins (CtHtrA and CtTsp) were generally observed to be greater when PBMC and either endometrial or endocervical cell co-cultures were stimulated (Figure 2) compared to each of the cell types cultured and stimulated separately. However, endocervical and endometrial cell co-cultures with autologous PBMC from two of the four participants showed this enhanced response to CtHtrA but not to CtTsp indicating that for each different participant primary reproductive epithelial cell culture differentially responds to different chlamydial PAMPs.

In three of the four primary cell cultures, the levels of IL-6 induced by the presence of live *Chlamydia* was not increased during the PBMC and endocervical or endometrial co-cultures compared to the individual epithelial or PBMC cell cultures and in some cases the co-culture of these cells actually showed a mildly reduced amount of IL-6 in response to live-*Chlamydia*. This is similar to the dichotomy in results found for HeLa and HEp-2 cells when co-cultured with THP-1 cells *in vitro* and stimulated with either the chlamydial proteins (CtHtrA and CtTsp) or live *Chlamydia*. Three of the four primary PBMC cultures were found to produce higher IL-6 levels in response to either the protein or chlamydial stimulants, than the corresponding participants’ endometrial or endocervical cells. The exception was tissue sourced from QUTPRT02, with by far the highest IL-6 levels following stimulation of endometrial and endocervical epithelial cells (approximately 10–100 fold higher).

### Participant peripheral blood mononuclear cells respond to chlamydial stress response proteases independent of disease cohort

**Figure 3:**
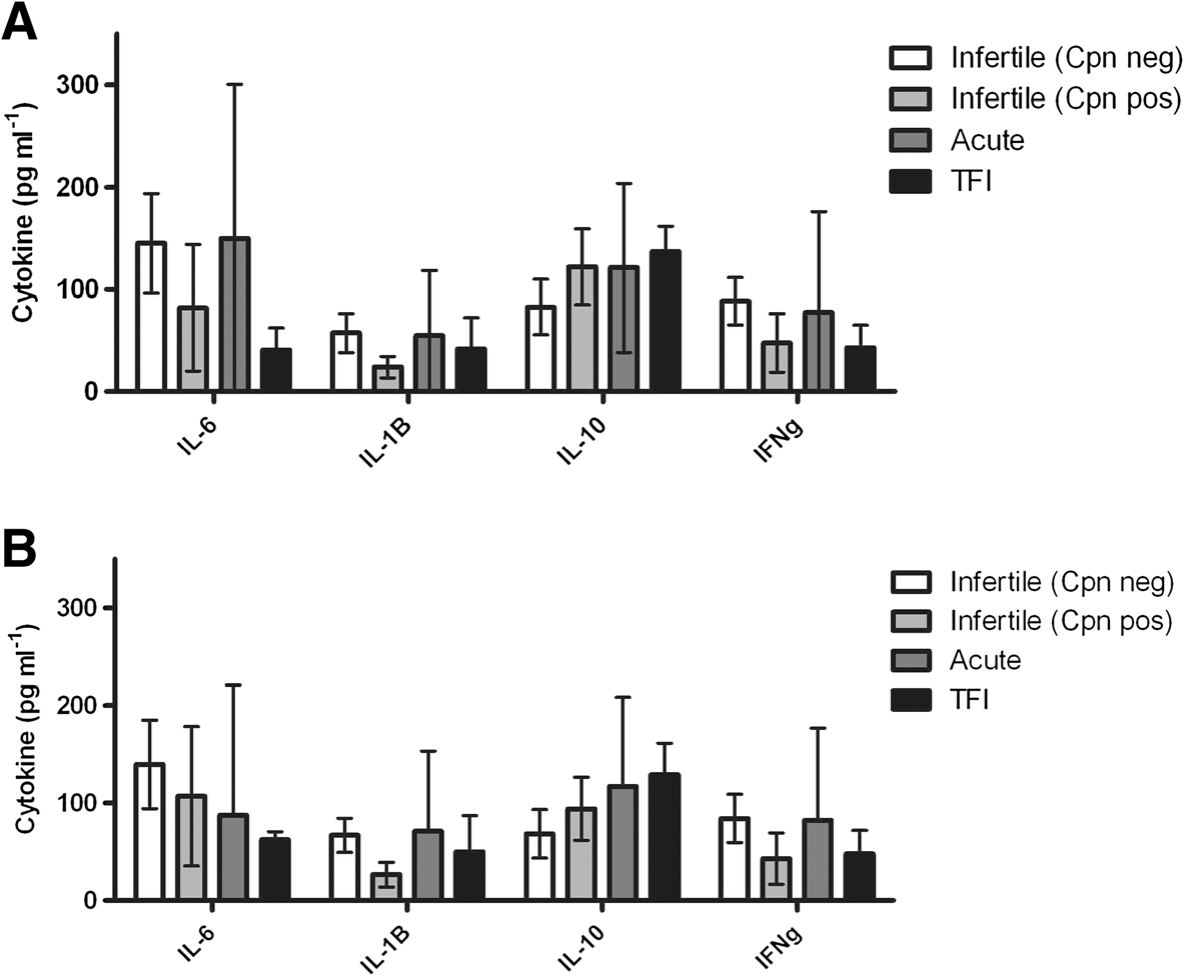
**Production of cytokines from PBMCs stimulated with CtTsp and CtHtrA according to participant cohort.** PBMCs were isolated from patients belonging to either Infertile (Cpn negative refers to serologically negative for *C. pneumoniae* IgG MEDAC ELISA), Infertile (Cpn positive or serologically positive for *C. pneumoniae* IgG MEDAC ELISA, Acute chlamydial infection or TFI cohorts. PBMCs were stimulated *in vitro* with **A)** CtTsp and **B)** CtHtrA, and IL-6 levels detected in supernatants after four days of culture. Unpaired two-tailed t-tests have been performed between cohorts and no significant differences were seen. The cohort numbers were: Infertile Cpn neg n = 7, infertile Cpn pos n = 4, acute infection n = 4, Tubal factor infertility n = 3.

In order to further understand the role PBMCs may have in the immune response to these chlamydial antigens, we examined a larger selection of participant PBMC responses to the antigens and *Chlamydia*. PBMCs from participants were isolated and stimulated with CtHtrA and CtTsp (previously described cohorts [11,24]), cytokines were measured at 96 h. The participants were grouped into the following disease cohorts; acute *C. trachomatis* infection, *C. trachomatis* tubal factor infertility, infertile *C. trachomatis* unrelated (*C. pneumoniae* serological status was tested and this cohort was split into two depending on this results). We also analysed cHSP60 and observed similar responses as previously reported [22,23,25]. We analysed all PBMC data using heatmaps clustered by cytokine and patient responses to observe correlated cytokine and participant (cohort) responses (Additional file 2: Figure S2). No significant difference was observed for the types of cytokines induced by either CtHtrA or CtTsp from the participants belonging to the different disease cohorts (Figure 3). Infertile women (not chlamydial induced) who had no serology against or reported history of *C. trachomatis* or *C. pneumoniae* infections produced the highest levels of cytokines in response to these proteins, including IL-6 (Figure 3). The range of concentrations of cytokines produced in response to these proteins was very broad in the acute infection cohort, suggesting that there may well be differential adaptive cellular immune responses to these proteins in this population which results in different levels of cytokine induction. However, PBMCs isolated from women who had not been exposed to either *C. trachomatis* or *C. pneumoniae* actually produced the highest levels of IL-6 providing further weight to the evidence that CtHtrA and CtTsp are PAMPs which induce an innate IL-6 response from both epithelial and mono-nuclear cells. There was a trend for the chlamydial tubal factor infertility cohort PBMCs to secrete lower concentrations of cytokines in response to CtHtrA and CtTsp, with the exception of IL-10. Other cytokines which were tested for but not detected in any patient cohort were IL-13, IL-12, and IL-4. PBMCs from the participant cohort with no prior chlamydial exposure showed a tendency towards a more inflammatory cytokine profile, with higher levels of IL-6, IL-1β and IFNγ. These data imply that CtTsp and CtHtrA have conserved PAMPs which are recognised by mononuclear cells and which could drive a pathological immune response.

### Induction of IL-6 by Chlamydia, and the secreted proteases CtTsp and CtHtrA involves MEK1/2 MAP kinases

The sustained induction of IL-6 by the epithelial cells observed here and the modulation of this in the presence of monocytic cells is similar to what has been reported for chronic inflammatory diseases of the gut, Crohns disease and ulcerative colitis. In these inflammatory diseases the intestinal epithelial production of IL-6 is increased by the presence of macrophages and CD4+ T-cells [26] and the presence of the pro-inflammatory cytokine IL-1β [27]. In order to understand if the IL-6 observed in these experiments similarly occurs as a consequence of prior secretion of IL-1β and the immune pathways involved, the laboratory model cultures of HeLa and HeLa co-culture with THP-1 were repeated using a variety of immune pathway modulators (24 h for IL-1β and 96 h for IL-6).

**Figure 4:**
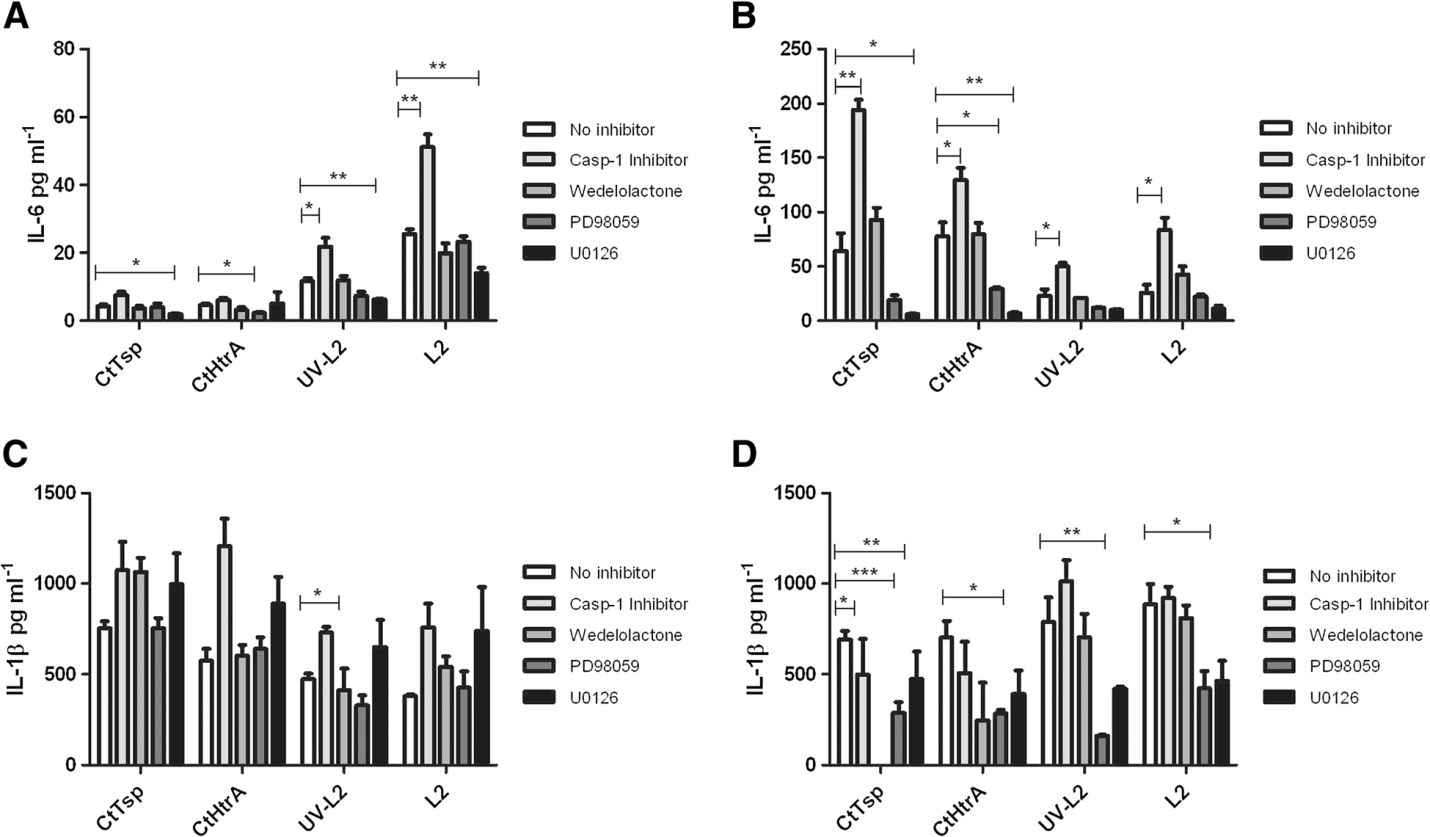
**Dependence of HeLa IL-6 and IL-1β response on cellular signalling pathways.** The secretion of IL-6 into the media from HeLa or HeLa with THP-1 cultures in response to the various stimulants is shown in the graphs. The IL-6 response at 96 hours is shown in Fig **A** and **B**. **A)** HeLa. **B)** HeLa with THP-1 co-culture. **C)** HeLa IL-1β response at 24 h and **D)** HeLa and THP-1 co-culture IL-1β levels at 24 h. (n = 3) Unpaired two-tailed t-tests have been performed between cohorts.

A caspase-1 inhibitor was tested because caspase-1 initially activates IL-1β as part of the inflammasome response (reviewed [28]). Inhibition of caspase-1 actually resulted in increased IL-6 production in response to all stimulants (Figure 4), but in HeLa only cultures there was no effect on the IL-6 secretion except in response to live *Chlamydia* where the levels also significantly increased (Figure 4). Wedelolactone inhibits IKK, a kinase involved the final stages of NF-κB activation cascade [27]. IKK inhibition did not alter the IL-6 levels secreted into the media under any of the culture conditions (Figure 4). PD98059 is a broad MEK inhibitor which results in decreased downstream JNK, STAT and p38 pathways induction (reviewed, [29]). U0126 inhibits MEK1/2, leading to decreased ERK1/2 signalling. Broad MEK inhibition did decrease the IL-6 secretion in response to CtHtrA, CtTsp, and live *Chlamydia* in the HeLa only cell culture (Figure 4). In the co-culture model, IL-6 secretion in response to CtHtrA and CtTsp was significantly reduced by broad MEK (PD98089) or MEK1/2 inhibition (U0126) (Figure 4).

In the co- culture model experiments (HeLa and THP-1) the production of IL-1β was significantly dependent on NF-κb (wedelolactone addition resulted in reduced IL-1β secretion) in response to both CtHtrA and CtTsp proteins. IL-1β secretion from HeLa and THP-1 co-cultures in the presence of the MEK inhibitors was also generally reduced (significantly in response to CtTsp, UV killed *Chlamydia* and live *Chlamydia*). Hence, the MEK pathways under which IL-1β secretion was reduced also showed a reduced IL-6, supporting that a higher IL-6 response may be preceded by Il-1 production. However, direct signalling to NF-κb also induced IL-1β (wedelolactone) and this pathway was not required for the secretion of IL-6, thus there are several distinct pathogen recognition pathways that can be activated by *Chlamydia* or chlamydial components to induce an IL-1β response.

### IL-6 and other pro-inflammatory cytokines are induced in mice reproductive tissues

The mouse model has been widely used to attempt to investigate the factors involved in chlamydial immunopathology. Interestingly, a previous investigation of IL-6 knockout mice did not find a difference in pathological outcome compared to the wild-type [30]. However, the IL-6 knockout mice are of a genetic background that includes C57BL/6 which are now known to have much less frequent development of pathology in response to *Chlamydia*[31]. Therefore, it is likely that an IL-6 knockout in a different genetic background may show a different result. We set out to validate that mouse epithelia also produce IL-6 in response to *Chlamydia* and the mouse *Chlamydia* (*Chlamydia (C.). muridarum*) homologous stress response proteases as a proof of concept for potential future IL-6 investigations in a different mouse model. These proteins are closely conserved between the two strains, with CtHtrA and CmHtrA sharing 96% identical amino acids (482/497) and 93% similarity (462/497), and CtTsp and CmTsp sharing 90% identity (580/642) and 95% (615/642) similarity of amino acid sequence. Firstly, we validated that the *C. muridarum* homologs induce an IL-6 response in the human cells. Ecc-1 cells were used for this initial comparison. There were no significant differences between CmHtrA and CtHtrA induced IL-6 secretion, and CmTsp induced higher levels of IL-6 than CtTsp (Additional file 3: Figure S3).

**Figure 5:**
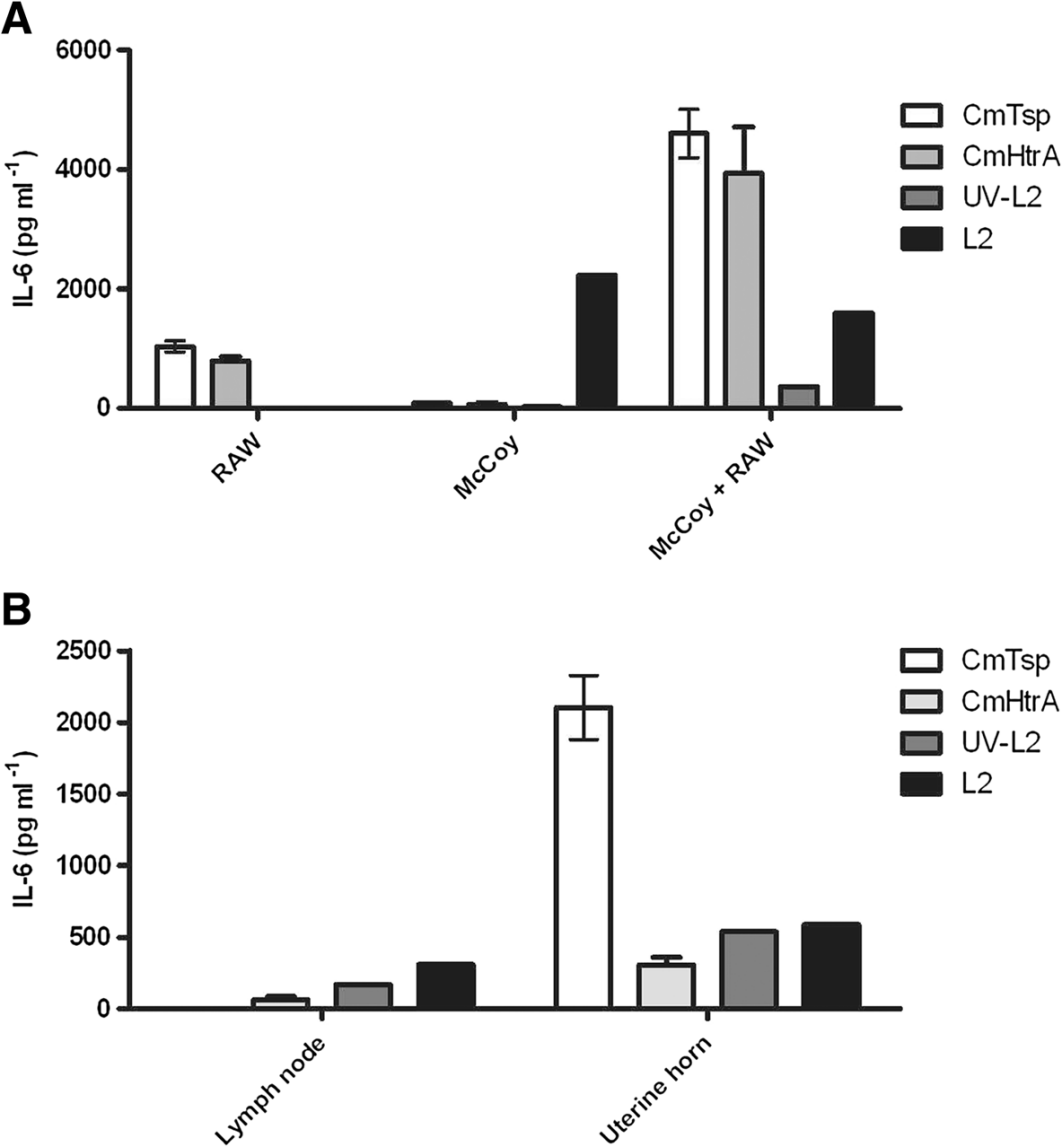
**Mouse cells produce IL-6 in response to the chlamydial stress response proteases and *Chlamydia*. A)** The figure shows the IL-6 production from mouse laboratory cell culture mono-layers with McCoy and co-culture with RAW264.7 cells in response to the stimulants. **B)** The graph shows the IL-6 production by primary uterine horn and caudal lymph node mouse cell culture in response to the stimulants. Stimulants are indicated to the right of each graph by the shaded bars (n = 2).

Mice fibroblasts (McCoy) and mice macrophages (RAW264.7) were then tested to ensure the *C. muridarum* proteins also induce IL-6 from mice cells. Other than IL-6 we were able to detect IL-10, GM-CSF, TNFα production in response to the proteins and *Chlamydia* (Additional file 4: Figure S4). None or physiologically irrelevant levels of cytokine was detected for IL-2, IL-4, IL-5, IL-6, IL-12, or IFN-γ under all conditions. The secreted levels of IL-6, IL-10, GM-CSF, and TNFα were all increased in the co-culture models compared to individual cell cultures when the stimulant was CmHtrA or CmTsp (Figure 5, and Additional file 4: Figure S4). However, for live *Chlamydia* the amount of IL-6 was reduced in co-culture models compared to the mono-cultures alone (Figure 5), consistent with what we already observed with the human cell culture models (Figures 1 and 2).

In order to assess if these observations reflect the local response in the mouse genital tract a primary culture model was conducted where uterine tissue and the local caudal lymph node from naïve mice was isolated, cultured and exposed to the stimulants and secreted cytokines measured, at 96 h. The predominant cytokine detected was IL-6 from both uterine horn and lymph node primary tissue in response to CmTsp, CmHtrA, live *Chlamydia* and UV killed *Chlamydia* (Figure 5B). GM-CSF was also detected to be produced in response to the proteins and *Chlamydia* stimulants by the primary culture of uterine horns. CmTsp induced strong IL-6 responses from both these tissues and the laboratory model cell cultures. IL-6 and IL-5 production was generally higher from lymph node tissues, whereas IL-6, IL-5, IL-10 and GM-CSF were higher from the uterine horn cell cultures. Therefore IL-6 produced by both human and mice species in response to their respective *Chlamydia* strains and two exported stress response proteases (Tsp and HtrA) may be a contributor to the innate cellular response to this pathogen and development of pathology.

## Discussion

This study has observed that the IL-6 response to *Chlamydia* and chlamydial PAMPs (in this case two stress response protease CtTsp and CtHtrA) varies widely in different reproductive cultures, which may implicate the level of IL-6 response as one of the factors which determines the disease outcome in women. The IL-6 was strongly induced by the proteases Ct/CmTsp and Ct/CmHtrA, live and UV killed *Chlamydia* in epithelial and mono-nuclear cell cultures. Live *Chlamydia* but not UV killed *Chlamydia* resulted in a reduced amount of IL-6 secreted when mononuclear and epithelial cells were co-cultured, suggesting that perhaps signalling for IL-6 induction may be yet another immune pathway for which *Chlamydia* has evolved a mechanism for immune-modulation. Secretion of IL-6 by epithelia and mononuclear cells in response to *Chlamydia* has been previously observed [32]. The co-culture based modulation of IL-6 has been previously observed by others at a day 3 time point following *Chlamydia* cultures in the presence of HeLa cells and co-cultures [20]. However, this is the first report of differential levels of IL-6 from primary human reproductive tissue and differential co-culture effects from human and animal models. The sustained nature of this response is also potentially important. Cytokines commonly reported in the literature has being detected at 24 and 48 h after chlamydial addition to PBMC [19,22,25], laboratory models or primary cultures were not detected at the 96 h time point, all though consistent with the previous literature when we did look for IL-1β at 24 h (as previously reported [19,21,25]) in our model we did detect this cytokine. Therefore, our model overall is consistent with previous findings, however, the extended time point we used could be important given the sustained presence of IL-6. This implies that IL-6 is a prolonged or sustained response to *Chlamydia* compared to many other cytokines, which is likely important in the disease setting.

Preliminary data presented here indicates that the IL-6 induction in response to the externally supplemented chlamydial stress response proteases and live chlamydial infections involved MEK pathways. The presence of IL-1β or GM-CSF along with (or prior to) IL-6 in the human or mouse culture models (respectively) suggests that IL-6 is involved in the innate pathological response to *Chlamydia*. Given that the IL-6 was detected in response to these antigens during primary cell cultures of reproductive epithelia from human participants, this data further supports the cellular paradigm of chlamydial disease pathology, that is the initial innate cellular response to the *Chlamydia*, and/or potentially exported chlamydial PAMPs such as CtTsp and CtHtrA can drive a pathological immune process leading to tissue damage, and IL-6 may be a cytokine involved in this disease mechanism. Both the sustained (until 96 h when most other cytokines were not detected) and the variability in concentration of IL-6 observed between different individuals also supports this possible role for IL-6 given that we know the infection outcome varies between individuals.

The induction of IL-6 was also detected when participant PBMCs were isolated and stimulated with the proteins or *Chlamydia*. Given that there were no significant cohort differences, it appears that these CtTsp and CtHtrA may encode conserved PAMPs able to be recognised by innate pathogen marker receptors on human immune and epithelial cells. The trend towards higher levels of inflammatory cytokines in previously unexposed patients appears to be consistent with the results seen in primary cell culture, where IL-6 but no IL-10, IL-4 or IL-5 was detected. Others have shown cohort-specific differences in cytokine response for different chlamydial PAMPs suggesting that some other chlamydial PAMPs drive an adaptive and pathological immune response. For example, PBMCs from *Chlamydia*-positive infertile women secreted more IL-6, IL-10 and IL-1β in response to Inc proteins than PBMCs from *Chlamydia*-positive fertile women [33]. The data presented here suggests that the co-interaction between epithelial and immune cells has a greater effect on immunopathological outcomes than monocytic or epithelial cell responses alone. Thus, measurement of cytokine release from stimulated PBMC may not sufficiently reflect the local cytokine milieu in the reproductive tract during infection. In particular the ability of live *Chlamydia* (but not UV killed) to reduce the IL-6 response during co-culture models may imply that IL-6 production is a host pathway targeted by this organism.

## Conclusions

We have identified a potential role for *Chlamydia* and chlamydial stress response proteases in the induction of differing levels of IL-6 from reproductive epithelia which may be involved in the damaging disease process. IL-6 was induced from both peripheral mononuclear cells and epithelial cells from primary human cultures with large variations in the concentration induced. IL-6 was also detected when the mouse homolog proteins were tested against primary mouse cell cultures. Combined these data suggest that IL-6 is induced during the innate response to *Chlamydia* and HtrA and Tsp. Therefore, the chlamydial stress response proteases HtrA and Tsp have conserved PAMPs which induce IL-6 and could be antigens which play a role in the development of disease pathology in some women. There was no evidence from the PBMC data of a cohort specific IL-6 response to CtHtrA or CtTsp. Therefore, the large variation in IL-6 response by the epithelial and un-exposed mononuclear cells to these antigens and the whole chlamydia suggests that there may be a pre-disposed likelihood of a high or low innate immune IL-6 response which may be an important factor in disease outcome from chlamydial infection. There were also different IL-6 responses during co-cultures with mononuclear cells from different participant epithelia or lab cell models compared to epithelia alone, in some cases the IL-6 response to *Chlamydia* was dampened by the co-culture, further supporting that the individual IL-6 response could be a major factor in the modulation of chlamydial infection disease outcome.

## Abbreviations

cHSP60: *Chlamydia* heat shock protein 60; CtTsp: *Chlamydia trachomatis* tail specific protease; CtHtrA: *Chlamydia trachomatis* high temperature requirement A; CmTsp: *Chlamydia muridarum* tail specific protease; CmHtrA: *Chlamydia muridarum* high temperature requirement A; PBMC: Peripheral blood mononuclear cells

## Competing interests

The authors declare that they have no competing interests.

## Authors’ contributions

KC designed and conducted some experiments, analysed data and drafted the manuscript, SS, PP, SM, and VK conducted some of the experiments and analysed data, JAA contributed to experimental design and participant recruitment, WMH contributed to conducting experiments, experimental design and analysis, and manuscript drafting. All authors have reviewed the manuscript. All authors have read and approved the final manuscript.

## Supplementary Material

Additional file 1: Figure S1 HeLa cell model including the cHSP60 induced cytokine response. Click here for file

Additional file 2: Figure S2 Cytokine secretion after stimulus with cHSP60 (A), HtrA (B) or TSP (C) for individual patient samples with hierarchical clustering of samples (x axis) and inflammatory cytokines (y axis). Patient cohort (infertile Cpn negative - light blue, Infertile Cpn positive - dark blue, acute - red, TFI - yellow) and cytokine type (humoral - black, cytotoxic - white) are denoted for each sample on the top and left axes, respectively. (D) cHSP60 cytokine response shown graphically as pooled cohort data, as shown for other proteins in Figure 3. Click here for file

Additional file 3: Figure S3 Comparison of mouse and human *Chlamydia* stress response proteases as antigens with Ecc1 cells. Click here for file

Additional file 4: Figure S4 Primary mouse tissue cytokine responses to the stimulants. Click here for file
