# Supplementary figures and images for: The IL-6 response to Chlamydia from primary reproductive epithelial cells is highly variable and may be involved in differential susceptibility to the immunopathological consequences of chlamydial infection
Source: BMC Immunol. 2013 Nov 15;14:50. doi: 10.1186/1471-2172-14-50 (PMC4225670; doi:10.1186/1471-2172-14-50)

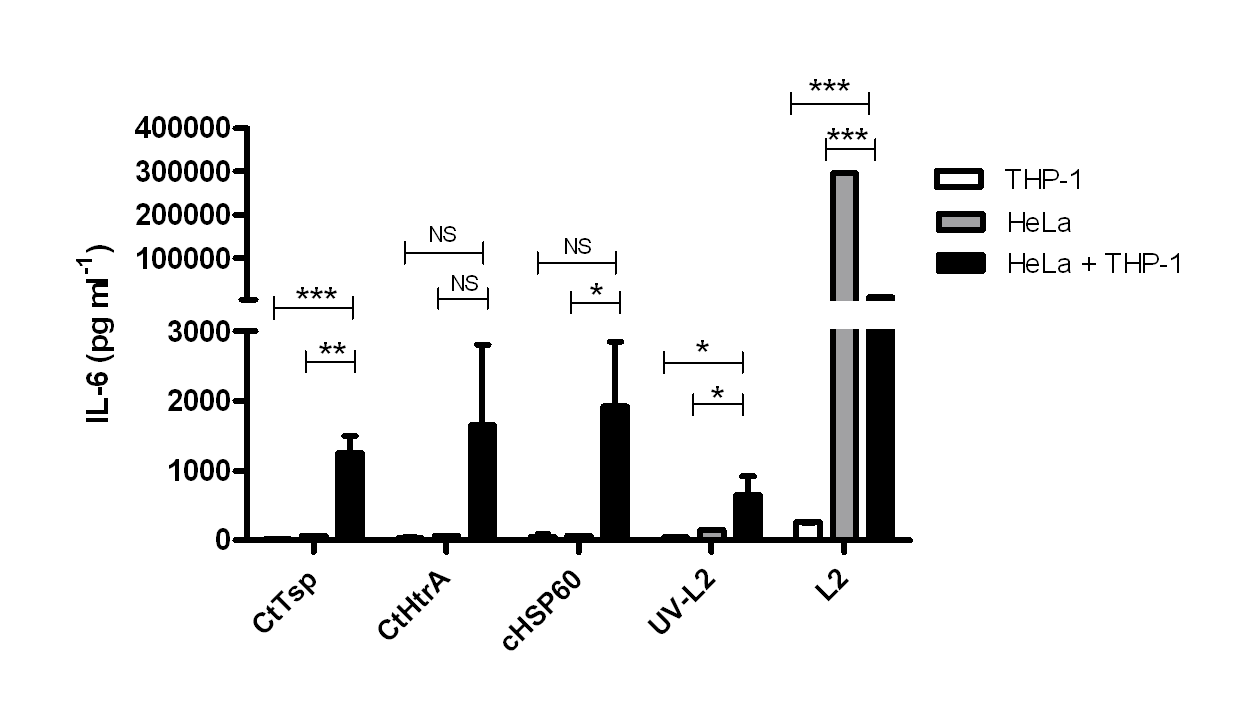

Supplement: Additional file 1: Figure S1 — HeLa cell model including the cHSP60 induced cytokine response. [file 1471-2172-14-50-S1.tiff]

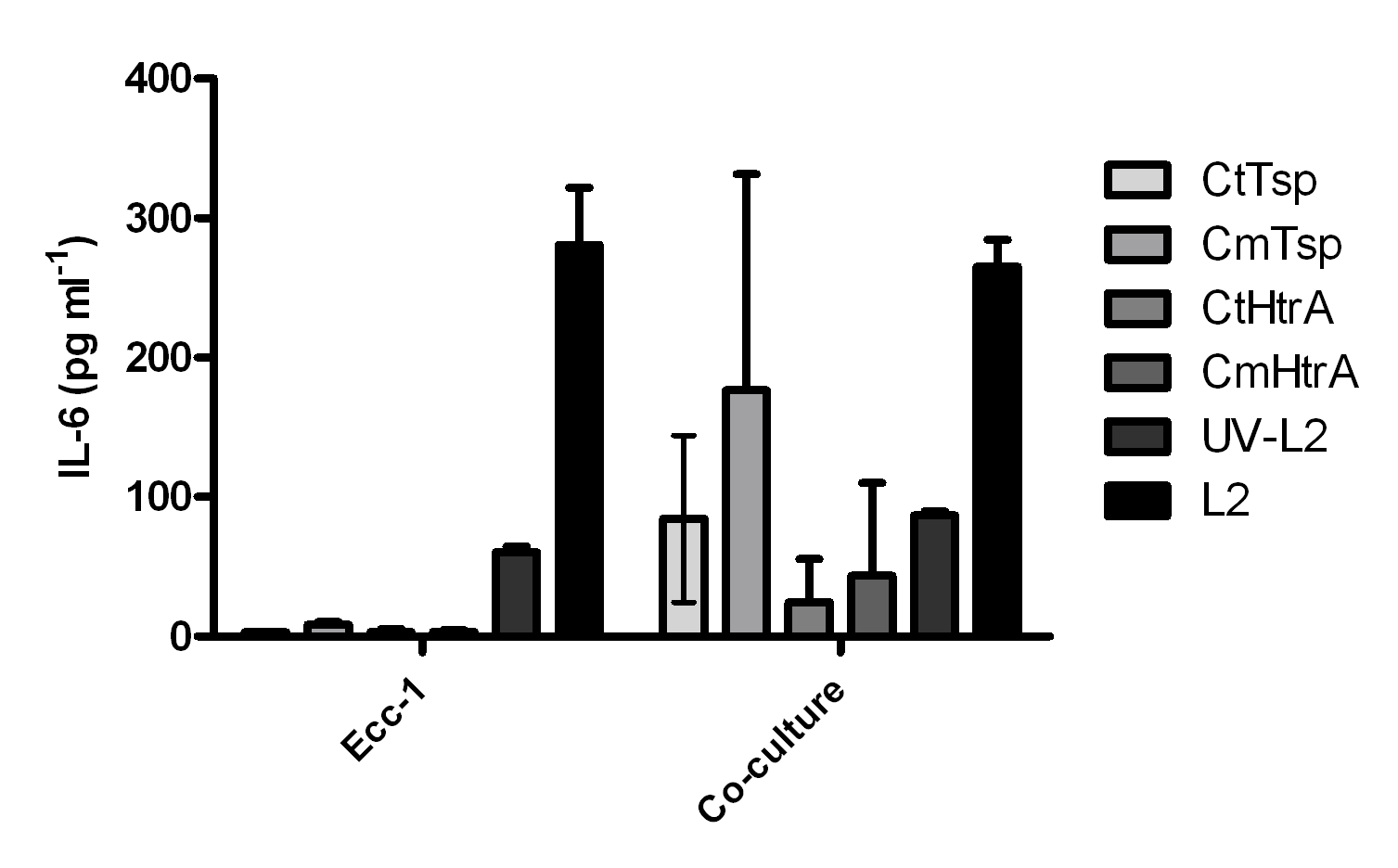

Supplement: Additional file 3: Figure S3 — Comparison of mouse and human Chlamydia stress response proteases as antigens with Ecc1 cells. [file 1471-2172-14-50-S3.tiff]

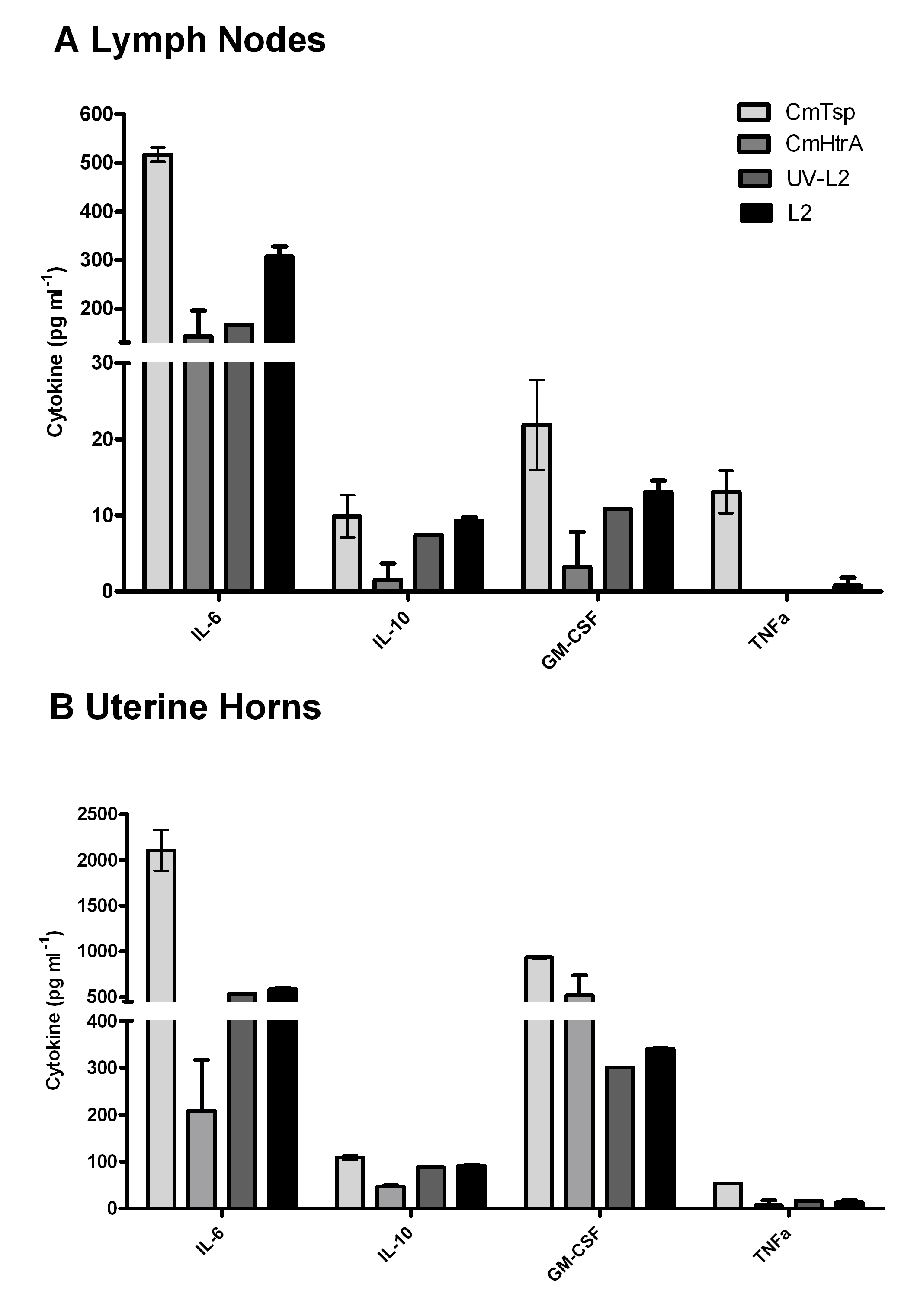

Supplement: Additional file 4: Figure S4 — Primary mouse tissue cytokine responses to the stimulants. [file 1471-2172-14-50-S4.tiff]
